# Resting-state brain activity and association with physical activity

**DOI:** 10.3389/fnagi.2026.1765112

**Published:** 2026-04-20

**Authors:** Georgia Koromila, Martin Dyrba, Dominik Wolf, Florian Fischer, Kristel Knaepen, Bianca Kollmann, Harald Binder, Andreas Mierau, David Riedel, Andreas Fellgiebel, Oliver Tüscher, Stefan Teipel, Sofia Faraza

**Affiliations:** 1 German Center for Neurodegenerative Diseases (DZNE), Rostock/Greifswald, Germany; 2 Faculty of Medicine, University of Thessaly, Larissa, Greece; 3 Department of Psychiatry and Psychotherapy, University Medical Center Mainz, Mainz, Germany; 4 Department of Psychiatry, Psychotherapy and Psychosomatic Medicine, University Medical Center Halle, Halle (Saale), Germany; 5 Institute of Movement and Neurosciences, German Sport University Cologne, Cologne, Germany; 6 Leibniz Institute for Resilience Research (LIR), Mainz, Germany; 7 Department of Neuropsychology and Psychological Resilience Research, Central Institute of Mental Health (ZI), Mannheim, Germany; 8 Institute of Medical Biometry and Statistics (IMBI), University Medical Center Freiburg, Freiburg, Germany; 9 Department of Exercise and Sport Science, Lunex International University of Health, Exercise and Sports, Differdange, Luxembourg; 10 Clinic for Psychiatry, Psychosomatics and Psychotherapy, AGAPLESION Elisabethenstift, Darmstadt, Germany; 11 German Center for Mental Health (DZPG), Site Halle-Jena-Magdeburg, Halle (Saale), Germany; 12 Institute of Molecular Biology (IMB) Mainz gGmbH, Mainz, Germany; 13 Department of Psychosomatic Medicine and Psychotherapy, University Medical Center Rostock, Rostock, Germany; 14 University Hospital of Child and Adolescent Psychiatry and Psychotherapy, University of Bern, Bern, Switzerland

**Keywords:** accelerometers, actigraphy, healthy aging, physical activity, resting-state functional connectivity

## Abstract

**Introduction:**

Normal aging is associated with alterations of functional connectivity in brain neuronal networks. Altered network connectivity may be associated with accelerated cognitive decline. Physical activity is considered a beneficial lifestyle factor for maintaining cognitive health. Higher intensities of physical activity may induce structural and functional changes in the brain, particularly in regions involved in cognitive functions. However, the underlying neural mechanisms are not widely investigated. Our aim was to examine the association between resting-state functional connectivity of brain networks previously associated with cognitive and motor functions, physical activity and cognitive performance in healthy older adults.

**Methods:**

We analyzed resting-state fMRI, physical activity and neuropsychological data of 149 healthy older adults (mean age: 68 years). Physical activity was measured by using actigraphs worn for 7 days and categorized into moderate-to-vigorous activity. Euclidean norm minus one values used to represent mean overall physical activity. We used a hypothesis driven seed-based approach and data-driven independent component analysis to examine brain network activity of *a priori* selected brain regions and networks.

**Results:**

No significant associations were found in the seed-based analyses. The independent component analyses showed spatially restricted effects of moderate-to-vigorous physical activity in frontal regions of the default mode and salience networks, at *p* < 0.01 uncorrected.

**Conclusion:**

Different physical activity intensities were not significantly associated with resting-state functional connectivity of various brain networks in a sample of healthy older adults. This finding contrasts with the results of previous cross-sectional studies.

## Introduction

Physical activity benefits cardiovascular health and promotes resilience against cognitive decline and dementia (McGregor et al., 2018; Sofi et al., 2011; Weuve et al., 2004; Yaffe et al., 2001), with recent evidence highlighting its neuroprotective effects, particularly in older adults (Buchman et al., 2019; Sofi et al., 2011).

Aging is associated with functional changes and alterations in functional connectivity (FC) of higher order networks, such as the default mode network (DMN), central executive network (CEN), dorsal attention network (DAN) and salience network (SAL) (Gogniat et al., 2022; Jockwitz and Caspers, 2021; Voss et al., 2016). Decreases in resting-state FC within the neural networks have been associated with cognitive deficits (Andrews-Hanna et al., 2007; Jockwitz et al., 2017; Salami et al., 2014; Shaw et al., 2015; Tsvetanov et al., 2016) and have been implicated in the risk of developing dementia and neurodegenerative diseases, such as Alzheimer’s disease (AD) (Hamer and Chida, 2009; Sofi et al., 2011). However, physical activity has been shown to significantly improve the FC of these networks (Domingos et al., 2021; Ferreira et al., 2022). Different types and intensities of exercise are shown to have different effects on connectivity within and between different brain regions (Domingos et al., 2021).

Higher levels of physical activity measured at baseline have been found positively correlated with an increased FC of DMN, SAL and left control network (Pruzin et al., 2022). Physical activity intervention studies have demonstrated higher FC within the anterior DMN and the DAN (Gogniat et al., 2022; Voss et al., 2016), the DMN and the SAL (Dion et al., 2021), and the DMN and the fronto-executive and fronto-parietal networks (Voss et al., 2010). On the other hand, decreased FC in networks, such as the DMN, the SAL, and left control network was strongly associated with sedentary time (Domingos et al., 2021; Vergoossen et al., 2021). FC of primary motor regions and DMN areas have also been associated with improved motor performance after physical activity interventions (Flodin et al., 2017; McGregor et al., 2018; Voss et al., 2010). In addition, CEN, which has been negatively associated with sedentary time, may be functionally enhanced by moderate-to-vigorous physical activity (Dion et al., 2021).

These data points to the importance of FC measures in the evaluation of the efficacy of physical activity in cognitive aging.

In the current study, we investigated the association between objectively measured physical activity and resting-state FC of brain networks in a sample of healthy older adults. Based on previous findings from cross-sectional studies (Gogniat et al., 2022; McGregor et al., 2018; Pruzin et al., 2022; Soldan et al., 2022), we hypothesized that higher FC of networks involved in cognitive and motor processes and shown to be functionally enhanced by physical activity interventions would be associated with higher physical activity at baseline. Specifically, we analyzed resting state FC of *a priori* selected brain networks and regions, such as DMN, SAL, CEN, visual (VN) and sensorimotor network (SMN). Physical activity intensities, such as the amount of moderate-to-vigorous activity (MVPA) and the overall physical activity, as represented by Euclidean norm minus one (ENMO) values, were objectively measured using actigraphs. Moreover, we investigated the association between FC of brain networks and habitual physical activity as measured by the Physical Actvity Scale for the Elderly (PASE) questionnaire (Washburn et al., 1993). Building on previous research that examined distinct associations between physical activity derived from accelerometers (Soldan et al., 2022; Voss et al., 2016) or from self-reports (Dorsman et al., 2020) with FC, we examined these associations by combining different metrics within the same cohort. Additionally, we examined covariates, such as sex, age, education, and cognitive performance of our sample. To our knowledge, the literature on the association between different physical activity aspects, such as frequency, duration and habitual activity, with FC of brain networks related to both cognitive and motor performance is limited. A combination of various aspects of physical activity may better identify which brain networks are related to habitual activity in healthy aging. This could contribute to the design of physical activity intervention or longitudinal studies aimed at understanding how lifestyle factors influence brain aging.

## Materials and methods

### Subjects and procedures

The data used in this article were obtained from the AgeGain study (Wolf et al., 2018), a longitudinal, interventional, multi-center, multi-modal imaging trial (German Clinical Trials Register, ID: DRKS00013077). The focus of the trial was to investigate the neurobiological mechanisms underlying cognitive and physical training in healthy older adults. The main exclusion criteria were the current –or history of– psychiatric, neurological, cognitive disease, brain lesions or any criterion that could affect MRI acquisition. A more detailed description of the study can be found in the study protocol (Wolf et al., 2018). All participants were enrolled by three recruiting centers in Germany: Mainz (University Medical Center Mainz, Department of Psychiatry and Psychotherapy), Rostock [University Medical Center Rostock, Clinic of Psychosomatic and Psychotherapeutic Medicine and German Center for Neurodegenerative Diseases (DZNE)] and Cologne (German Sport University Cologne, Institute of Movement and Neurosciences). The participants were recruited by local newspaper announcements and flyers.

For the purposes of the current study, we selected a subset of the full longitudinal dataset based on the availability of accelerometric and resting state fMRI data. Specifically, we used physical activity, neuropsychological data, and resting-state fMRI data from 149 participants, as measured at baseline level.

### Physical activity measurements

Actigraphs, namely the portable wristband (GeneActive, Kimbolton, United Kingdom), worn for 7 days, were used to objectively measure physical activity. The minimum h of weartime was set to 16 h per day as a criterion. These devices are validated (Esliger et al., 2011) and were found to be comparable to the well-established ActiGraph (ActiGraph, Pensacola, United States) (Hildebrand et al., 2014). The wristbands used a three-axis accelerometer, a heat flux sensor, a galvanic skin-response sensor, a skin-temperature sensor and a near-body ambient temperature sensor to capture data for 1 week (Wolf et al., 2018). The raw data was extracted by GENEActiv Software Version 3.2 as provided by the manufacturer and all subsequent analyses were performed using R package GGIR version 1.9-1 (Migueles et al., 2019) in RStudio Version 1.0.136 (R Studio Team, 2015 Integrated Development for R. RStudio, Inc., Boston, MA [Computer Software v0.98.1074]). These included auto-calibration (van Hees et al., 2014) extraction of the Euclidean Norm minus one (ENMO), a metric of the overall acceleration signal subtracted by the gravitational component (van Hees et al., 2013), identification and imputation of potential non-wear time as well as calculation of time spent at moderate/vigorous activity levels (Migueles et al., 2019). This level was defined such that 80% of 5 s segments of 60 s bouts had to exceed the ENMO threshold of 100 mg (Menai et al., 2017). ENMO was used as a representation of overall physical activity, and it was measured in milligravity (mg). Moderate-to-vigorous physical activity was measured in minutes/day (min/d) to reflect time spent at higher physical activity levels.

Moreover, we selected data using the Physical Activity Scale for the Elderly (PASE) (Washburn et al., 1993). The PASE is a validated 12-item self-administered questionnaire that assesses activities typically chosen by older adults, such as walking, exercise, housework, yardwork or caring for another person. It examines the frequency, duration and intensity level of the activity, with scores ranging from 0 to 793, with higher scores indicating greater physical activity.

### Neuropsychological assessment

Our participants completed a battery of neuropsychological tests. For the current analyses, we selected cognitive tests representing memory, working memory and executive functions from the full neuropsychological battery (Wolf et al., 2018) and we extracted composite scores. More specifically, memory and working memory were measured by the Verbal Learning Memory Test (VLMT), a German version of the auditory verbal learning test (Helmstaedter et al., 2001) and by subtests of the Wechsler Memory Scale- revised (WMS-R): digit span, block span (forward and backward) and visual paired associations (Wechsler, 1987). Executive functions were assessed by Trail Making Test B (Reitan, 1956), Tower of London (Shallice, 1982), Leistungsprüfsystem (LPS) (comparable to Raven Matrices): subtest 4 (LPS-4: logical reasoning) and 8 (LPS-8: visuospatial perception) (Horn, 1962). Including the abovementioned neuropsychological tests, we built composite scores for the three cognitive domains: Memory, Working memory and Executive functions. This was realized by standardizing all raw variables as T-scores based on distribution parameters of the neuropsychological assessment and then calculating the mean score of each time point of the assessment. The neuropsychological scores used as covariates in our statistical models.

### MR data acquisition

The scans were acquired from three 3T-MRI Siemens scanners (1 Verio, 1 Prisma, 1 Trio) using identical acquisition parameters and harmonized instructions. T1-weighted anatomical images were captured using Magnetization Prepared Rapid Gradient Echo (MPRAGE) sequence with the following parameters: sagittal slices = 176, scan time = 4.18 min., repetition time (TR) = 1,900 ms, echo time (TE) = 2.45, flip angle = 9°, field of view (FOV) = 250 mm, voxel volumes = 1.0 × 1.0 × 1.0 mm. Resting-state functional MRI (rs-fMRI) scans were acquired with the following parameters: scan time = 11.02 min., transversal slices = 60, slice thickness = 2.5 mm, TR = 1,000 ms, TE = 30.6 ms, flip angle = 56°, FOV = 210 mm. Participants were instructed to keep their eyes closed without thinking of anything in particular or falling asleep.

### MR data preprocessing

The resting state-fMRI scans were processed using the Data Processing Assistant for Resting-State fMRI (DPARSFA, Version 4.3, Chao-Gan and Yu-Feng, 2010), implemented in MATLAB (MATLAB, Version 2020a, “MathWorks—Makers of MATLAB and Simulink,” MathWorks, 2024). After the removal of the first 10 volumes of each fMRI scan, a series of steps were applied, such as slice timing correction and realignment to the mean volume. To reduce the influence of noise, the following aspects were regressed out for the seed-based analysis: linear trend, 12 motion parameters, white matter, cerebrospinal fluid, and the global signal as nuisance regressors. For the independent component analysis (ICA) this step was omitted. The functional images were filtered with a band-pass filter between 0.01 and 0.1 Hz. The T1-weighted MPRAGE scans were co-registered to the mean fMRI images, segmented into white matter, gray matter and cerebrospinal fluid, and spatially normalized to Montreal Neurological Institute (MNI) space using the Diffeomorphic Anatomical Registration Through Exponentiated Lie Algebra (DARTEL) algorithm (Ashburner, 2007). The resulting non-linear deformation fields were then applied to the frequency-filtered fMRI data to transform them to MNI reference space. Finally, fMRI scans were resampled to 3 mm × 3 mm × 3 mm voxel size and smoothed with a 6 mm full-width-at-half-maximum (FWHM) Gaussian kernel.

### Seed-based global functional connectivity

We conducted seed-based connectivity analysis using 19 *a priori* selected seed regions of interest (ROIs) representative of DMN, CEN, SAL, VN and SMN based on previous literature (Chang et al., 2019; Li et al., 2023; Mayka et al., 2006; Seeley et al., 2007; Shen et al., 2019; Weiler et al., 2014; Table 1). Seed-based connectivity (SBC) maps were obtained, representing the functional activation of each seed region. This resulted respectively in 19 SBC maps. Subsequently, we performed global FC analyses to measure the strength of FC of the regions of interest. We aimed to examine the global FC of each region separately. To perform these analyses we used a script from Franzmeier et al. (2017) which included the following steps: (1) we obtained a correlation of each voxel’s resting-state time series with those of the seed-regions, (2) the correlations were transformed to Fisher Z values, thresholded at *z* > 0.3 and averaged to produce a global FC value. Only positive correlations were used, since they indicate higher connectivity strength (Cole et al., 2012).

**TABLE 1:** Montreal neurological institute (MNI) coordinates of 19 a priori regions of interest.

Study	Seed-network	ROI name	MNI coordinates (x, y, z)
Li et al. (2023)	CEN	Anterior cingulate cortex	2, 36, 22
Weiler et al. (2014)	CEN	Dorsolateral prefrontal cortex (left)	−45, 18, 48
Weiler et al. (2014)	CEN	Dorsolateral prefrontal cortex (right)	45, 18, 48
Li et al. (2023)	DMN	Inferior parietal lobules (left)	−50, −63, 32
Li et al. (2023)	DMN	Inferior parietal lobules (right)	48, −69, 35
Li et al. (2023)	DMN	Medial prefrontal cortex (left)	−2, 58, −6
Li et al. (2023)	DMN	Medial prefrontal cortex (right)	3, 54, −2
Li et al. (2023)	DMN	Posterior cingulate cortex (left)	0, −52, 26
Chang et al. (2019)	SAL	Ventral striatum (superior)	10, 15, 0
Seeley et al. (2007)	SAL	Dorsal anterior cingulate cortex	10, 34, 24
Chang et al. (2019)	SAL	Ventral striatum (inferior)	9, 9, −8
Mayka et al. (2006)	SMN	Lateral premotor cortex	−26, −6, 56
Mayka et al. (2006)	SMN	Supplementary motor area	−3, 6, 53
Mayka et al. (2006)	SMN	Sensorimotor cortex	−39, −21, 54
Mayka et al. (2006)	SMN	Supplementary motor area	−2, −7, 55
Shen et al. (2019)	VN	Dorsal visual network (left)	−37, −79, 10
Shen et al. (2019)	VN	Dorsal visual network (right)	38, −72, 13
Shen et al. (2019)	VN	Primary visual network	2, −79, 12
Shen et al. (2019)	VN	Ventral visual network	0, −93, 4

CEN, Central Executive Network; DMN, Default Mode Network, SAL, Salience Network; SMN, Sensorimotor Network; VN, Visual Network.

### Data analysis

#### Physical activity measurements across sites

We conducted ANOVA analyses using R (R Foundation for Statistical Computing, Vienna, Austria., R core team, 2023) to determine if there were differences in the measured physical activity intensities between the three study sites.

#### Physical activity and seed-based global functional connectivity

Linear regression analyses were performed to examine the cross-sectional relationship between physical activity intensities and global FC of the 19 seed regions. MVPA, ENMO and PASE were used as predictors of FC of the selected 19 seed regions. Regression models were adjusted for sex, age, education, baseline neuropsychological performance and site (model: network connectivity ∼ physical activity intensities + covariates).

#### Physical activity and independent component analysis

In addition, we conducted a data-driven approach using the resting-state fMRI data obtained from the experimental group. The extraction of brain networks was carried out using ICA (Beckmann et al., 2009) as implemented in MELODIC (Multivariate Exploratory Linear Decomposition into Independent Components), toolbox of FSL (FMRIB Software Library, Version 6.0, Oxford, United Kingdom).^1^ The resulting 30 components were visually inspected to identify brain networks based on previous literature (Cao et al., 2016; Habas et al., 2009; Heine et al., 2012; Rosazza and Minati, 2011; Seeley et al., 2007). FSL’s dual regression generated subject-specific versions of the ICA maps and associated time-series to derive subject-level resting state FC z-maps. The group-average set of spatial maps was regressed into each subject’s 4D dataset. This resulted in a set of subject-specific time-series. Next, these time-series were regressed into the same 4D dataset, resulting in a set of subject-specific spatial maps (Beckmann et al., 2009; Filippini et al., 2009; Nickerson et al., 2017). To define the functional activation areas which belong to the corresponding resting-state networks, network-specific masks from the ICA maps of the whole sample, thresholded at *p* < 0.05 TFCE (Threshold-Free Cluster Enhancement), were obtained. In addition, templates of brain networks, as previously identified by Shirer et al. (2012) were used for a more precise identification of the components. We regressed each contrast of interest (e.g.. physical activity intensities, sex, age, education, neuropsychological performance, site) on the respective resting-state functional network. We then tested voxel-wise for statistically significant differences across subjects using FSL’s randomize permutation-testing tool (Winkler et al., 2014). The resulting t-test statistic images (tstat) were thresholded at *p* < 0.01, uncorrected for multiple comparisons, for each contrast.

## Results

### Demographics and physical activity results

Demographic characteristics of the total sample and across three study sites and actigraphy results are shown in Tables 2, 3. Our sample consisted of 149 participants with a mean age of 68.2 years (SD = 5.7), and slightly more than half (52.3%) were female. On average, participants had completed 15.8 years of education (SD = 2.6). Participants spent on average 34.2 min/day on moderate-to-vigorous activity over the 7-day period. The overall physical activity, as measured by ENMO, was 27.8 mg/day. In addition, participants spent 469.2 min/day inactive. The PASE results showed an average score of 151.8.

**TABLE 2:** Demographic characteristics and actigraphy data (*N* = 149).

Variable	Mean (SD) [min, max]
Age	68.2 (5.7) [60–88]
Sex (male: female)	71:78
Education	15.8 (2.6) [9- –21]
ENMO (mg)	27.8 (7.4) [13.44—49.76]
MVPA (min/day)	34.2 (21.2) [1.89- –104.36]
Inactivity (min/day)	469.2 (81.2) [277—685.7]
PASE	151.8 (64.1) [30—350]

ENMO, Euclidean Norm Minus One; MVPA, Moderous-to-Vigorous Physical Activity; PASE, Physical Activity Scale for the Elderly.

**TABLE 3:** Demographic characteristics and actigraphy data across sites.

Variable	Site 1 (Mainz)	Site 2 (Rostock)	Site 3 (Cologne)
*N*	34	48	67
Sex (male: female)	15:19	16:32	40:27
Age (SD)	69.7(5.9)	68.4 (6.4)	67.2 (4.8)
Education (SD) ENMO (SD) MVPA (SD) Inactivity PASE	15.3 (2.8) 24.8 (5.9) 26.5 (16.4) 469.7 (59.6) 135.6 (66.6)	15.8 (2.1) 27.8 (7.2) 34.04 (23.0) 453.6 (79.9) 168.98 (66.8)	16.0 (2.8) 29.6 (7.7) 38.8 (21.0) 481 (91.1) 148.2 (58.2)

ENMO, Euclidean Norm Minus One; MVPA, Moderous-to-Vigorous Physical Activity; PASE, Physical Activity Scale for the Elderly.

### Site differences

The ANOVA results showed a significant difference between the study sites for ENMO [*F*(2, 148) = 4.5, *p* < 0.05, η^2^ = 0.06] and MVPA [*F* (2,148) = 3.6, *p* < 0.05, η^2^ = 0.05]. A Tukey *post-hoc* test revealed that the ENMO scores for site 3 (Cologne) (Δ = 4.6, SE = 1.5, *p* < 0.05) were significantly different from the scores for site 1 (Mainz). Similarly, *post-hoc* analyses showed that the MVPA scores for site 3 (Δ = 11.7, SE = 4.3, *p* < 0.05) were significantly different from those of site 1. Descriptive statistics indicated that ENMO scores were higher for site 3 (*M* = 29.4, SD = 7.7) than site 1 (M = 24.8, SD = 5.9). Similarly, MVPA scores were higher for site 3 (*M* = 38.2, SD = 21.2) than for site 1 (*M* = 26.5, SD = 16.4).

### Physical activity and seed-based global functional connectivity

The linear regression analyses did not show any significant association between global FC of the 19 *a priori* selected seed regions and the physical activity intensities. One of the statistical models showed a significant association between the PASE score and the Supplementary motor area [β = 0.17, SE = 0.08, *t*(129) = 2.05, *p* = 0.042)] indicating that higher scores on PASE were associated with higher global FC of the Supplementary motor area. In the linear models, neuropsychological performance was used as a covariate and some of them showed an association between global FC of the inferior parietal lobule and working memory performance, as well as between the primary visual network and memory performance (*p* < 0.05) (Results in Supplementary Table 1).

### Physical activity and independent component analysis

**FIGURE 1:**
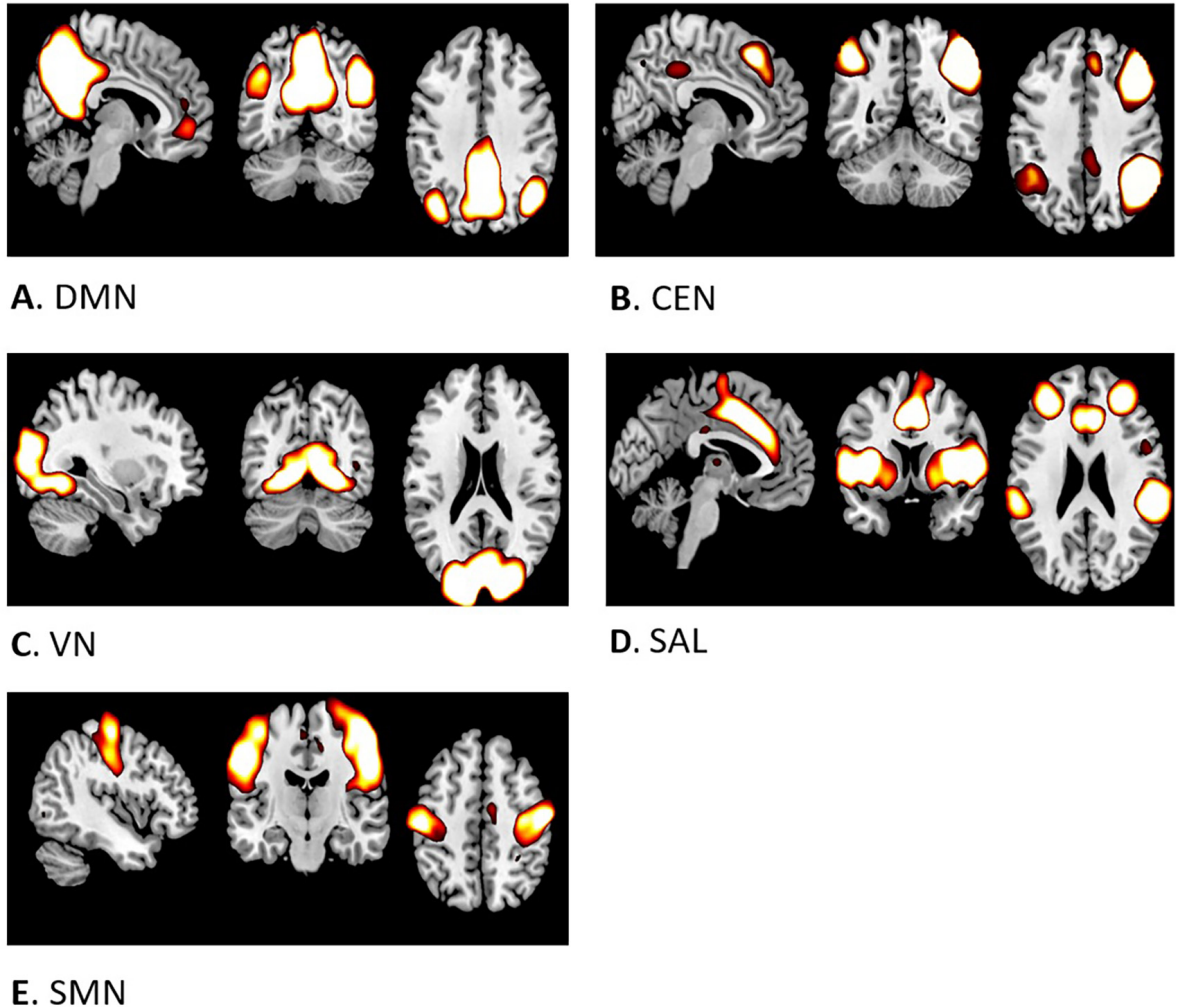
Spatial maps of selected networks: **(A)** Default Mode Network, **(B)** Central Executive Network, **(C)** Visual Network, **(D)** Salience Network, **(E)** Sensorimotor Network.

**FIGURE 2:**
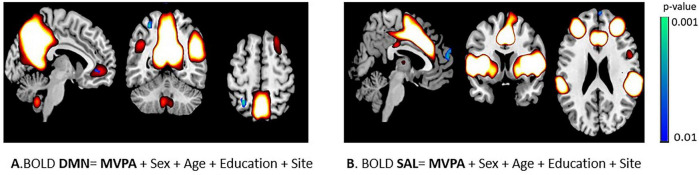
Yellow-white voxels represent the functional networks of **(A)** Default Mode Network (DMN) and **(B)** Salience Network (SAL). Blue voxels indicate significant association between network connectivity and moderate-to-vigorous activity (MVPA) at *p* < 0.01 uncorrected. Association was restricted only to the corresponding networks only by network-specific masks obtained from the independent component maps from the whole sample thresholded at *p* < 0.05 TFCE, one-sample *t*-test.

ICA spatial maps obtained from the whole sample corresponded to DMN, CEN, VN, SAL and SMN (Figure 1). Results from dual regression showed spatially restricted associations between MVPA and DMN and SAL connectivity, at *p* < 0.01, uncorrected (Figure 2). No significant results were found for the ENMO and functional network connectivity.

## Discussion

In this study, we examined the cross-sectional associations between physical activity intensities and resting-state FC at baseline in a sample of healthy older adults. Physical activity was measured objectively under normal living conditions over a 1-week period, which has the advantage over self-reported measures by avoiding reporting bias. However, we also examined physical activity by using the PASE questionnaire to better capture habitual aspects of physical activity.

We investigated FC using two methodological approaches. First, we performed seed-based analyses by examining the global FC of 19 *a priori* selected seed regions. Our results showed no significant association between global FC and physical activity intensities. Second, we extracted brain networks using a data-driven approach. Our results showed effects of MVPA on frontal regions within the DMN and SAL network, although spatially restricted. Even though the effects of physical activity on the FC are spatially restricted, we observed an overlap in frontal activations across the two networks. However, these findings are not robust enough to draw a conclusion about a cross-sectional relationship between MVPA and FC of DMN and SAL. Our findings are consistent with those of Voss et al. (2016), who measured physical activity in a large sample of healthy older adults (*N* = 189) using actigraphs for 7 days and found no association between MVPA and FC of networks, such as the DMN, CEN, VIS, and SAL. Furthermore, they suggested that cardiorespiratory fitness, rather than physical activity, is related to FC. However, despite the conceptual similarity to this study, there are some methodological differences. First, we used wrist-worn accelerometers unlike Voss et al. (2016), who used hip-worn accelerometers. Differences in placement may influence estimates of minutes spent in walking or being sedantary (Kerr et al., 2017). Second, we used different coordinates to define our ROIs representing large-scale networks. Moreover, our findings contradict those of other studies that used actigraphs to measure physical activity and examined functional activations. For instance, Dion et al. (2021) found a positive association between MVPA and FC of CEN in a small group of 18 non-demented older adults and Gogniat et al. (2022) demonstrated a positive association between physical activity and FC of DMN in frontal and parietal areas of 47 older adults. Additionally, Soldan et al. (2022) showed an association between higher total volume of physical activity within the DMN in a sample of 136 healthy older adults. Similarly, Callow et al. (2024) found an association between a greater total volume of physical activity and high FC within the DMN in a sample of 116 non-demented older adults. Furthermore, Pruzin et al. (2022) showed an association between higher physical activity and higher FC in the DMN, SAL, and left control networks of 167 healthy older adults. The discrepancy in our results may be due to the small sample sizes in the studies of Dion et al. (2021) and Gogniat et al. (2022), which allow for a Type I error. Furthermore, these studies used different methods to assess physical activity, such as mean (Dion et al., 2021) or average (Gogniat et al., 2022) steps per minute, steps per day (Pruzin et al., 2022) or total volume of physical activity (Callow et al., 2024; Soldan et al., 2022). Another possible explanation for the lack of association could be the *a priori* selection of the ROIs based on literature research, which may differ from other studies. For instance, Gogniat et al. (2022) defined DMN using seeds of posterior cingulate gyrus and superior frontal gyrus, which differ from our defined ROIs for the DMN, namely the posterior cingulate cortex, medial prefrontal cortex and inferior parietal lobules.

Some of the linear models showed an association between high global FC of the left inferior parietal lobule and high performance on working memory tasks. This is consistent with previous studies (Baldo and Dronkers, 2006; Chochon et al., 1999; Greene and Killiany, 2010). Baldo and Dronkers (2006) showed that patients with damage to the inferior parietal lobule were impaired in their ability to repeat words, digits and sentences while Chochon et al. (1999) and Greene and Killiany (2010) showed that subregions of the inferior parietal lobules were associated with performance on tasks involving numbers, such as the Digit Symbol and Trail-Making-Test B. Moreover, some of the models showed an association between high global FC of the primary visual network and high performance on memory tasks. There are no robust findings across studies that support this association. Most of the studies restrict their analyses of memory performance to other networks, such as the DMN or CEN (Waner et al., 2023) or when including visual networks, do not focus on memory measurements (Khalilian et al., 2024). Since the functional activity of these regions is only associated with high cognitive performance and not physical activity, it raises questions about cognitive reserve and its association with lifestyle factors. Cognitive reserve is influenced by multiple factors beyond physical activity, such as education or occupation (Conti et al., 2021). It is possible that compensatory mechanisms may support cognitive performance independently of physical activity levels. However, the effects of physical activity may not be fully captured by static resting-state FC measures, but rather, they may be captured via vascular or neurobiological mechanisms (Nicolini et al., 2021). Therefore, these findings should not be interpreted as evidence that cognitive reserve is independent of physical lifestyle factors, but rather that the relationship is complex.

In addition, one of the models showed an association between high global FC of the supplementary motor area and the PASE score. To our knowledge, there is no evidence in the current literature linking the PASE score with the FC of the supplementary motor area. Most of the studies focus on different brain networks, such as Dorsman et al. (2020), who found no significant association between DMN activity and PASE. A possible explanation for our findings could be that the PASE measures activities related to balance, walking and muscle strength, in which the supplementary motor area is involved. Previous studies showed that the supplementary motor area is related to habitual aspects of motor behavior, such as self-initiated, voluntary movements, motor planning or preparation of coordinated movements (Jacobs et al., 2009; Nachev et al., 2008; Welniarz et al., 2019). This may explain the observed association with PASE, which may better capture habitual physical activity patterns than accelerometers. Moreover, it is plausible that regular engagement in daily physical activity of the participants could have enhanced neuronal signaling leading to high FC on motor networks (Seidler et al., 2015). This finding may also be interpreted as bidirectional, suggesting that participants with preserved FC on the sensorimotor regions are more likely to engage in daily physical activity. However, we must consider that PASE is a self-reported instrument and may not accurately reflect everyday physical activity levels. Furthermore, the lack of associations between FC of the supplementary motor area and MVPA or ENMO may reflect the fact that these metrics capture intensity and duration, but not qualitative aspects of physical activity, such as motor complexity, that are relevant to the supplementary motor area. Additionally, since habitual movement patterns represent complex behaviors, they are unlikely to be fully captured by isolated FC measurements. They may be more reliably reflected in multimodal approaches that integrate structural, functional and microstructural markers (Salvan et al., 2021). Future studies therefore should examine additional brain regions within motor networks, as well qualitative aspects of physical activity to better understand the relationship between habitual physical activity and brain function (Pesce, 2012).

Overall, our findings do not indicate associations between FC and objectively measured physical activity intensities. In the current study, we focused on static FC, as our primary aim was to investigate average connectivity patterns across the resting-state scans. However, we acknowledge that a dynamic FC may better capture the temporal flexibility of functional networks and could be sensitive to lifestyle-related effects, such as exercise, as it reflects changes in FC patterns over seconds or minutes across a scan (Nguyen et al., 2025). A dynamic FC could potentially reveal spatially or temporally hubs of plasticity that are not detectable by a static FC (Park et al., 2017). However, further investigation is required to determine whether these temporal fluctuations reflect underlying neural processes. In addition, a fine-grained parcellation allows for more detailed mapping of functional networks and may detect spatially localized connectivity patterns that may be not captured by a seed-based of data-driven approaches (de Reus and van den Heuvel, 2013). A static FC approach, which we used in the current study, could have missed significant localized effects within brain areas associated with exercise-related neuroplasticity. However, we should consider that dynamic connectivity analyses and fine-grained parcellation are more sensitive to noise, motion and require longer scan durations, which increases methodological complexity (Hutchison et al., 2013). In the present study, we used static FC as a robust and validated measure of average network organization. Future studies should consider different approaches to analyzing functional resting-state data, such as dynamic FC, fine-grained parcellation, or a combination of different approaches, to gain additional insight into the functional brain networks.

Another possible explanation for the lack of effects might be the fact that our participants were shown to be inactive for most hours of the day. This was evident from the actigraphy results. The actigraphy results showed that our participants were inactive for an average of 469.2 (81.2) min/day. The average level of inactivity is higher than the average levels of MVPA which were 34.2 (21.2) min/day and ENMO which were 27.8 (7.4) mg/day. However, we should note that a classification of low-active participants should be reserved for the following reasons. Our results are lower compared to those of Brady et al. (2023), in which older adults spent in average 77.6 min/day in MVPA. However, compared to other studies, our participants spent slightly more minutes per day on MVPA. For instance, Chen et al. (2025) showed that 191 older adults spent 21.5 (18.9) min/day on MVPA across 7-day period. In the study of Ramires et al. (2017), 971 older adults spent in average 11 min/day on MVPA (5 min bout) and 21.7 mg was the mean overall physical activity. Several other studies used different metrics, such the average steps per day, which makes the comparison of the activity levels difficult. In addition, the PASE results showed an average score of 151.8 (64.1). Given that the highest possible score is 793, a score of 151.8 seems to be not very high. However, according to the normative data of community-dwelling older adults the mean scores were 142.9 (age ≤ 70 years) and 110.8 (age > 70 years) (Washburn et al., 1993) which are slightly lower than our score. Moreover, other studies have reported an average PASE score of 126.2 (Dorsman et al., 2020) or 147.9 for males and 110.5 for females (Logan et al., 2013). Based on previous evidence, we can conclude that our average PASE score is representative or slightly higher than that of other healthy older adults. This may partially explain the association between higher FC of supplementary motor area and PASE scores, as discussed earlier. However, considering that there is no established cut-off score and that the standard deviation in our PASE score is 64.1 points, we cannot determine whether the participants were highly active in their daily lives. Similarly, given the heterogeneous literature on ENMO and MVPA, it is difficult to classify the participants as low- or high-active. In addition, the observed variability in both the standard deviation and range of measurements indicates that a clear floor effect is unlikely, although it cannot be entirely excluded.

Although accelerometers provide an objective measure of physical activity, it is important to note that several differences in the literature on activity levels have been observed. These differences may be due to variations in the definition of a bout, the thresholds for MVPA or the placement of the accelerometers. For example, Ramires et al. (2017) applied different bout criteria (non-bouted, 1, 5, and 10-min bout) and set a threshold of 100 mg to estimate MVPA, whereas Chen et al. (2025) set a threshold of ≥ 2,020 counts/min. In our study, we defined 1 min bout that had to exceed the threshold of 100 mg. Furthermore, Voss et al. (2016) and Dion et al. (2021) used hip-worn accelerometers, whereas other studies (Chen et al., 2025; Gogniat et al., 2022; Pruzin et al., 2022) used waist-worn. Our study, like others, used wrist-worn accelerometers (Callow et al., 2024; Ramires et al., 2017; Soldan et al., 2022). A systematic review by Gall et al. (2022) revealed discrepancies between wrist and hip accelerometers, suggesting that wrist placement overestimates MVPA and underestimates sedentary time. These discrepancies range from minutes to hours. It is important to consider these differences when we compare the physical activity outcomes of the present study to those of previous studies.

Additionally, it should be considered that there were differences across the three study sites in the ENMO and MVPA outcomes. Our results showed that the participants in Cologne had higher scores than those in Mainz. This could be explained due to different built environment of each location, such as walkability, cyclist infrastructure or sport facilities (Pedersen et al., 2022). This variability across sites may not reflect underlying neural mechanisms and could therefore increase noise in the data. This is a consideration which may partially explain the absence of significant associations. However, we should note that we used the site as a covariate in our analysis, and we did not find any significant associations.

The current research has several limitations to consider. First, the seed-based approach has the advantage of *a priori* selection of regions, but it limits the exploration of other brain regions. This is why we complemented an ICA approach. However, the ICA approach has the limitation of being a data-driven approach. The brain networks were extracted from the whole sample and were identified based on visual inspection and template matching (Shirer et al., 2012). This could mean that the identified regions of the networks might differ slightly from other studies. In addition, the statistical maps were thresholded at *p* < 0.01 without correction for multiple comparisons. This threshold was chosen to increase sensitivity, but it does not control the family-wise-error rate. This may increase the likelihood of false-positive findings. Third, the cross-sectional measurements preclude any conclusions about habitual physical activity levels and functional brain connectivity.

## Conclusion

Our findings indicate that different intensities of physical activity, such as MVPA and ENMO, were not significantly associated with the resting-state FC across brain networks, including DMN, CEN, SAL, SMN, VN in a sample of healthy older adults. These results differ from previous research and may reflect differences in FC measurements, sample characteristics or the cross-sectional design of the study. Moreover, our findings demonstrated an association between self-reported habitual physical activity and FC of the supplementary motor area. Our work extends the existing literature by considering different physical activity intensities, multiple brain regions and networks and various methodological approaches. Future research should focus on measuring physical activity over a longer period to gain a better understanding of the relationship between physical activity and resting state FC and to investigate different brain regions and functional activation patterns. Furthermore, future studies should incorporate multimodal imaging approaches and focus on the qualitative aspects of physical activity, such as coordination, motor complexity or cognitive engagement, to better understand its effects on brain function and increase sensitivity to detect associations.

## Data Availability

The datasets presented in this article are not readily available because we did not obtain consent from research subjects or the ethics committee to generally share the data with the public at the time when the study was approved. The data are available to researchers involved with the consortium within the scope of shared research projects. Requests to access the datasets should be directed to oliver.tuescher@uk-halle.de; flfische@uni-mainz.de.

## Footnotes

**footnote:** 1 https://fsl.fmrib.ox.ac.uk/fsl/docs/index.html
