# Supplementary material for: Resting-state brain activity and association with physical activity
Source: Front Aging Neurosci. 2026 Apr 20;18:1765112. doi: 10.3389/fnagi.2026.1765112 (PMC13136082; doi:10.3389/fnagi.2026.1765112)
Supplement: Supplementary file 1 [file Table_1.docx]

**Supplementary Table 1**

Results from linear regression models using physical activity intensities and neuropsychological scores

| Models | Estimate | *SE t* | 95% CI | | *p* |
| --- | --- | --- | --- | --- | --- |
|  |  |  | *LL* | *UL* |  |

**Model 1**
FC of inferior parietal lobule~ MVPA + WM + covariates 0.20 0.08 2.51 0.04 0.37 0.013

**Model 2**
FC of primary visual network~ MVPA + MEM + covariates 0.17 0.08 2.14 0.01 0.34 0.034

**Model 3**

FC of inferior parietal lobule~ ENMO + WM+ covariates 0.20 0.08 2.50 0.04 0.36 0.013

**Model 4**

FC of primary visual network~ ENMO + MEM+ covariates 0.18 0.08 2.15 0.01 0.34 0.033
________________________________________________________________________________________________________________

FC= functional connectivity, MVPA= moderate-to-vigorous activity, ENMO= Euclidean norm minus one,
WM= working memory, MEM= memory
